# Cerebral venous sinus thrombosis with head trauma and myeloproliferative neoplasm-unclassifiable: A case report

**DOI:** 10.1097/MD.0000000000043012

**Published:** 2025-08-29

**Authors:** Qiongxian Chu, Zhiwei Zhou, Hongyan Zhou, Zucai Xu, Ping Xu, Zhongxiang Xu

**Affiliations:** a Department of Neurology, Affiliated Hospital of Zunyi Medical University, Zunyi, China.

**Keywords:** anticoagulant, cerebral venous sinus thrombosis, etiology, head trauma, unclassifiable myeloproliferative neoplasm

## Abstract

**Rationale:**

Cerebral venous and sinus thrombosis (CVST) is a rare and special type of cerebrovascular disease characterized by complex etiology, diverse forms of onset, nonspecific clinical manifestations, and difficult diagnosis. Myeloproliferative neoplasm–unclassifiable (MPN-U) refers to clinical manifestations, laboratory findings, and morphological features that are consistent with the diagnosis of myeloproliferative neoplasms, but do not meet further specific classification criteria, or have overlapping features of 2 or more myeloproliferative neoplasms. Here, we report a case of CVST with MPN-U and a history of head trauma. This case report emphasizes the need to recognize such manifestations to avoid misdiagnosis and ensure timely treatment.

**Patient concerns:**

This case report presents a cerebral venous sinus thrombosis with head trauma and an unclassifiable myeloproliferative neoplasm male patient who experienced visual impairment with hearing loss, cerebration trauma.

**Diagnoses:**

CVST with MPN-U.

**Interventions:**

The anticoagulant treatment with low molecular weight heparin were administered in the hospital, then rivaroxaban was given after discharge.

**Outcomes:**

His blurred vision improved, and the sense of tightness in the parietal and occidental skin relieved at discharge. He took rivaroxaban 10 mg orally once a day after discharge. At 1-month follow-up, the blurred vision of this patient had significantly improved.

**Lessons:**

It is unique that CVST with blurred vision as the main clinical manifestation and 2 possible causes, including head trauma and MPN-U. For patients with CVST, in addition to anticoagulant therapy, it is also necessary to identify the etiologies.

## 1. Introduction

Cerebral venous and sinus thrombosis (CVST) is a rare disease that mostly occurs in young and middle-aged women, accounting for 0.5% to 1% of cerebrovascular diseases.^[1,2]^ CSVT obstructed the blood flow in cerebral sinuses and veins, resulting in intracranial hypertension and stroke, leading to long-lasting neurological consequences, even endanger life in severe cases.^[3]^ The CVST patients frequently showed headache, blurred vision, vomiting, and epilepsy on admission.^[3]^ The risk factors of CVST include systemic and focal infections, hereditary thrombophilia (V-factor Leiden mutation, protein C, and S deficiencies), and acquired prethrombotic state, which included pregnancy, postpartum, postoperative, head trauma, tumors, and oral contraceptives.^[4]^ Furthermore, previous studies suggested that myeloproliferative neoplasms (MPNs) were associated with CVST, especially polycythemia vera.^[5]^ MPN-unclassifiable (MPN-U) refers to clinical manifestations, laboratory findings, and morphological features that are consistent with the diagnosis of MPNs, but do not meet further specific classification criteria, or have overlapping features of 2 or more types of MPNs.^[6]^ Currently, there are no reports on the relationship between MPN-U and CVST. We report a case of CVST with MPN-U and a history of head trauma.

## 2. Case presentation

A 52-year-old male was admitted to our hospital due to paroxysmal visual impairment with hearing loss for 1 year and worsening for 2 months. One year ago, the patient fell down from the stairs (about 2 m high) and fell into a coma, and woke up several hours later without nausea, vomiting or blurred vision, he was diagnosed as cerebration trauma, left top epidural hematoma, occidental subdue hematoma, and left temporal mastoid fracture at the other hospital. Fortunately, paroxysmal binaural blurred vision accompanied by decreased hearing in both ears occurred, the above symptoms mostly occurred during standing up from a supine position and exercising, and lasting about 1 minute and gradually relieved, without fever, headache, nausea, vomiting, seizure, limb weakness and altered mental status. His binaural blurred vision and hearing loss have worsened significantly in the past 2 months, and there has been a sense of tightness in the parietal and occidental skin.

At admission, he was conscious, pupils of equal size and diameter (about 4 mm), sensitive capillary light reflex, normal binocular movement, visual acuity decreased in both eyes, more severe in the left side, decreased hearing in both ears. The volume, tone, and strength of muscle were normal, the tendon reflexes of upper and lower limbs were symmetrically elicited, physiological reflexes existed, and bilateral pathological signs were negative. Soft neck and negative meningeal irritation sign.

Blood routine showed hemoglobin 184.0 g/L (normal range, 130–175), crematoria 0.54 L/L (normal range, 0.4–0.5), mean corpuscular volume 104.1 AL (normal range, 80–100), mean corpuscular hemoglobin 35.7 pg (normal range, 27–34), erythrocyte counts, white blood cell counts, platelet counts, serum biochemical, thyroid-stimulating hormone, serum free T4, serum free T3, and erythromycin were normal. d-dimer 0.51 Ag/mL (normal range, <0.5). Erythrocyte sedimentation rate, C-reactive protein, rheumatoid factor, ANA + antinuclear antibody spectrum, anti-eutrophic cytoplasmic antibody, hypertrophy receptor antibody, immunoglobulin antibody, thyroid oxidase antibodies, hepatitis C virus, human immunodeficiency virus, trans-pacific partnership agreement, quantitative determination of immunoglobulin and related antigen of male tumor were negative. The pressure of the lumbar puncture cerebrovascular fluid was 310 mm H_2_O. The routine and biochemical of the cerebrovascular fluid (CSF) were normal. Recombination aquamarine 4, glacial fibrillate acidic protein, and myelin basic protein antibodies were negative in serum and CSF, myelin oligonucleotide lactoprotein antibodies was positive in the serum (niter 1:10) and negative in CSF.

**Figure 1.**
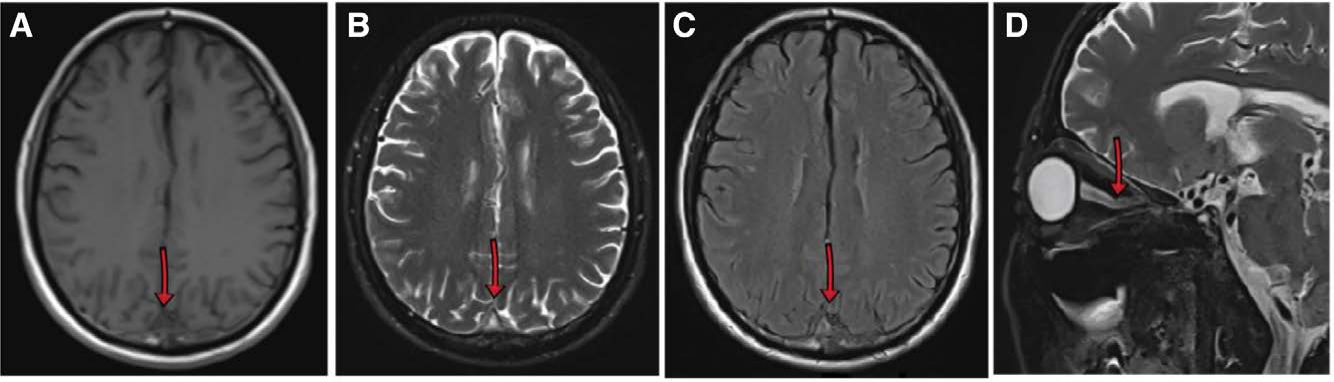
Neuroimaging and ophthalmologic findings of the patient. Cerebral MRI showed hyperintensity in the superior sagittal sinus on T2WI and FLAIR, with hypointensity on T1WI, and scattered hyperintense lesions in bilateral cerebral white matter on T2WI and FLAIR, with hypointense on T1WI (A–C). Orbital MRI showed the left optic nerve slightly thickened and hypointense in the near orbital segment on T2WI (D). FLAIR = fluid-attenuated inversion recovery, T2WI = T2-weighted images.

**Figure 2.**
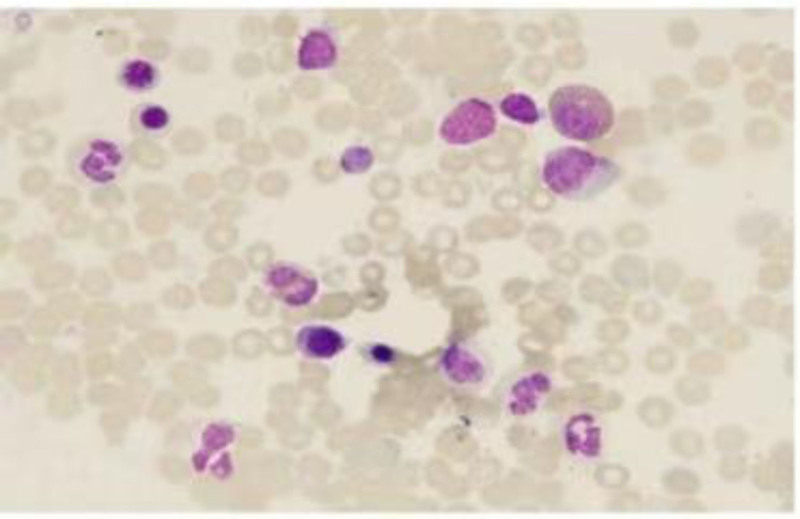
The morphology of bone marrow cells showed that the nucleated cells proliferated actively, the ratio of granulosa and red was 3.97:1, and mature red blood cells were distributed in a stacked pattern, consistent with the signs of polycythemia.

**Figure 3.**
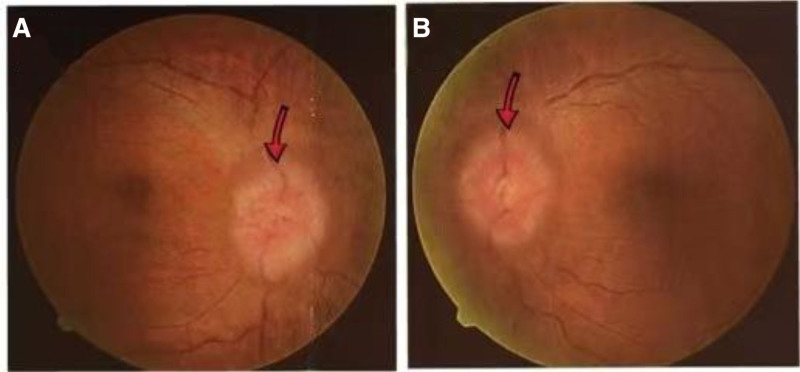
Bilateral fundus photography demonstrated bilateral papilledema (A, B).

**Figure 4.**
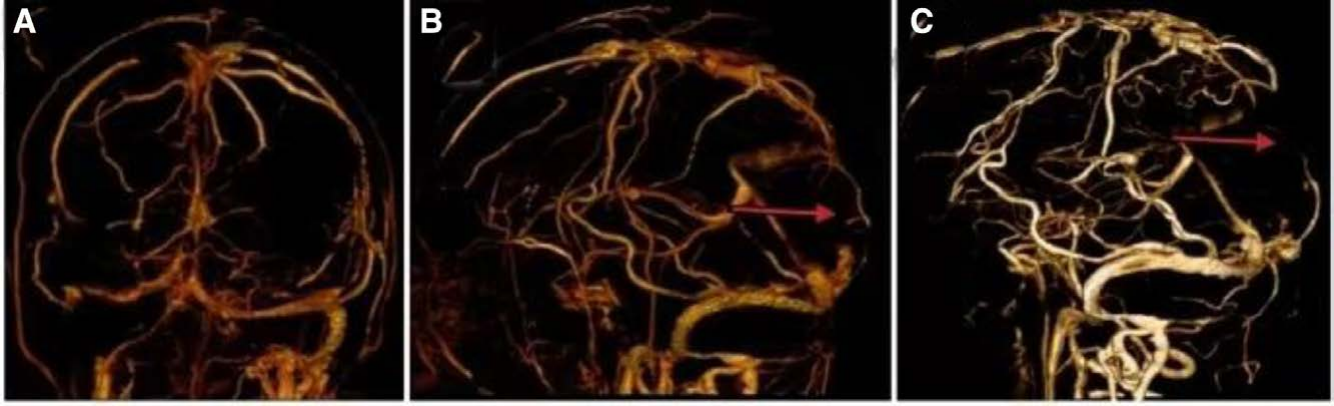
Computed tomographic venography (CTV) indicated that no contrast filling in the superior sagittal sinus, CVST was considered, the right transverse sinus and sigmoid sinus were small and locally discontinuous, anatomical variants were considered (A, B). The follow-up cerebral CTV showed no contrast-filled superior sagittal sinus (C). CVST = Cerebral venous sinus thrombosis.

Cerebral magnetic resonance imaging (MRI) showed hyper intensity in the superior digitalis sinus on T2-weighted images (T2WI) and fluid-attenuated inversion recovery (FLAIR), with hypo intensity on T1WI (Fig. 1A–C). Orbital MRI showed the left optic nerve slightly thickened and hypo intense in the near orbital segment on T2WI (Fig. 1D). Cervical, thoracic, and lumbar vertebra MRI with gadolinium enhancement revealed that cervical, thoracic, and lumbar degenerative changes. The morphology of bone marrow cells showed that the mature red blood cells were distributed in a stacked pattern, consistent with the signs of hypoglycemia (Fig. 2). The pathology of bone marrow biopsy exhibited onomatopoetic tissue accounted for about 40%, erythrocyte cells slightly increase, and prokaryote was about 1/high power field. The granulates were mainly in the middle and late juvenile stage, the erythrocyte cells were mainly in the middle late juvenile stage, and scattered prokaryote, mainly mature ambulated giant cells. MPN related genes (Janus kinase 2, myeloma preoperative leukemia, clonal expansion in nonproliferation neoplasms, granulate colony-stimulating factor receptor) and CRABLIKE P210, P190, P230 genes were negative in bone marrow. Bilateral fund photography demonstrated bilateral papilloma (Fig. 3A, B). Visual field examination revealed that the decreased light sensitivity in the central field of the left eye with circular visual field defect, and the decreased light sensitivity in the central field of vision of the right eye with peripheral dark spots. Brain–stem auditory evoked response (Bakers) on both left and right sides were abnormal (more pronounced on the left side). Visual evoked potential displayed the left visual evoked potential was significantly abnormal, while the right visual evoked potential was slightly abnormal. Cardiac and abdominal ultrasound, and electroencephalogram findings were normal. Computed tomographic venography (CTV) revealed segmental non-filling specifically in the posterior third of the superior sagittal sinus (extending from the coronal suture to the torcular herophili), accompanied by corresponding venous collateral formation. These radiographic findings were consistent with segmental cerebral venous sinus thrombosis. CVST was considered (Fig. 4A, B). There was no abnormality in the jugular veins.

Low molecular weight heparin 0.4 mL was injected subcutaneously every 12 hours for anticoagulant treatment, and intravenous infusion of manifold and glycerol fructose were administered to reduce intracranial pressure. Nine days later, repeated blood routine exhibited that hemoglobin 182.0 g/L, crematoria 0.52 L/L, mean corpuscular volume 104.0 AL, mean corpuscular hemoglobin 34.7 pg, d-dimer 0.40 Ag/mL. In the repeated lumbar puncture, the pressure of CSF was 200 mm H_2_O, CSF routine and biochemical were normal. The follow-up cerebral CTV showed no contrast-filled superior digitalis sinus (Fig. 4C). His blurred vision improved, and the sense of tightness in the parietal and occidental skin relieved at discharge. He took riverbank 10 mg orally once a day after discharge. At 1 month follow-up, the blurred vision of this patient had significantly improved.

## 3. Discussion and conclusions

Our patient presented with blurred vision as the main clinical manifestation, MRI suggests a slight thickening and abnormal signal in the near orbital segment of the left optic nerve, which was suspected to be optic neuritis. Concurrently, central nervous system delineating disease should be considered, especially myelin oligonucleotide lactoprotein associated disorders, which can present as various clinical manifestations. Further examination of serum and cerebrovascular fluid revealed negative recombination aquamarine 4, glacial fibrillate acidic protein, and myelin basic protein antibodies. The serum myelin oligonucleotide lactoprotein antibodies was positive, but the niter was very low at 1:10, indicating insufficient evidence for the diagnosis of myelin oligonucleotide lactoprotein associated disorders. During the diagnostic workup, we found the patient had intracranial hypertension, bilateral papilloma, impaired visual field, increased hemoglobin and elevated d-dimer, hyper intense in the superior digitalis sinus on T2WI and fluid-attenuated inversion recovery, combined with a history of head trauma, the diagnosis of superior digitalis sinus thrombosis was confirmed by brain CTV. Cervical, thoracic, and lumbar spine MRI excluded intracranial hypertension caused by spinal canal occupancy.

CVST is a rare but potentially life-threaten cerebrovascular disease, with an annual incidence rate of 5 cases per million.^[2]^ The clinical process of CVST is usually sub acute, the most common symptom is severe headache, which occurs in over 90% of cases.^[1,3]^ Clinical symptoms are related to the location, nature of thrombosis and the degree of secondary brain injury. The most common thrombosis site is superior digitalis sinus.^[2]^ The main manifestations are headache, seizures, paralysis, papilloma, and altered mental state.^[1]^ CVST occurs infrequently in general population and lack of specific manifestations are related to delayed diagnosis, and is easily confused with other cerebrovascular diseases.^[1]^ When the thrombus is located in the superior digitalis sinus, a “triangle sign” can be displayed on brain computed tomographic (CT), when it is located in the cortex or deep vein, a “ cord sign” can be displayed on brain CT, cerebral CT can also reveal complications of CVST, such as arachnophobia or parenthetical hemorrhage, cerebral edema, and ventricular enlargement or displacement.^[1]^ The occlusion of the affected venous sinus, irregular stenosis, and filling defects can be seen on magnetic resonance ethnography or CTV.^[1]^ DSA is the gold standard for diagnosing CVST and should be used when considering cardiovascular treatment.^[2]^ When CVST was suspected, abnormal signal in venous sinus on conventional MRI and elevated d-dimer may provide a clue.^[4]^

Nonproliferation neoplasms are clonal onomatopoetic stem cell diseases, with a reported 44% and 30% probability of thrombosis at diagnosis and after diagnosis.^[5]^ Our patient had elevated hemoglobin and crematoria, without cause of secondary hypoglycemia, phenomenally. Gene mutations in Janus kinase 2 V617F, CRABLIKE were not found. His diagnosis was the nonproliferation neoplasm-classifiable (NPN-C), which may be related to CVST. The mechanism of NPN-C leading to CVST may be similar to hypoglycemia Hera, with abnormally increased red blood cells, elevated blood viscosity, reduced blood flow velocity, and easily activated platelets leading to thrombosis.^[7]^

CVST was initially believed to occur in severe head injury patients with skull fractures adjacent to the aural venous sinus.^[8]^ However, CVST cases have also been found in patients with closed head injuries.^[8]^ More than one-third of patients with severe traumatic brain injury have CVST. Meanwhile, CVST must be considered even after minor head injury.^[9]^ The pathogenesis of CVST in trauma is not well-established, proposed hypotheses include sinus compression and thrombosis caused by skull fractures, intracranial anathemas or edema, endothelial injury of venous sinus, intramural hemorrhage at the site of venous drainage, sinusoidal rupture, thrombosis expansion of injured emissary veins, and post-BI coagulator eliciting the release of anticoagulant.^[9,10]^

Anticoagulant therapy with low molecular weight heparin or counteraction heparin is safe in the early stage of the disease, and can benefit even with hemorrhagic infarction, warfarin or the novel oral anticoagulants (transmigrate, riverbank, and appendix) can be used.^[2,11]^ If there is no new bleeding within 48 to 72 hours after head trauma, anticoagulant therapy can be initiated for patients with combined brain injury.^[8,12]^ Generally, the duration of anti coagulation was 3 to 12 months. The CVST patients with ineffective anti coagulation or concomitant cerebral hemorrhage can choose intramuscular intervention treatment, which included venous sinus contact thrombolytic, mechanical thrombolytic, balloon catheter thrombolytic, and venous sinus stent angioplasty.^[1,2,4]^ Clinically stable NPN-C does not require treatment yet, but follow-up and observation are needed.^[13]^ In our case, the symptoms were improved after subcutaneous injection of low molecular weight heparin. After discharge, the patient continued to take riverbank. Although the patient has a long course of disease, the blurred vision has significantly improved at the 1 month follow-up, and anticoagulant treatment was effective.

In summary, it is very rare that CVST with blurred vision as the main clinical manifestation and 2 possible causes which including head trauma and NPN-C. The clinical manifestations of CVST are usually atypical, and easily misdiagnosed. Early diagnosis and treatment usually play a crucial role in improving prognosis. The absence of headache—a hallmark symptom present in approximately 90% of CVST cases—posed a significant diagnostic challenge in this patient. This atypical presentation underscores the importance of maintaining a high index of suspicion for CVST even in the absence of cephalalgia, particularly when other neurological manifestations are present. For patients with CVST, in addition to anticoagulant therapy, it is also necessary to identify the causes, and actively intervene in the preventable ones to reduce the risk of recurrent CVST.

## Acknowledgments

We thank the patient included in this research.

## Author contributions

**Data curation:** Qiongxian Chu, Zhiwei Zhou, Hongyan Zhou.

**Formal analysis:** Zhongxiang Xu.

**Methodology:** Zucai Xu, Zhongxiang Xu.

**Resources:** Zucai Xu.

**Supervision:** Ping Xu, Zhongxiang Xu.

**Writing – original draft:** Qiongxian Chu, Zhiwei Zhou.

**Writing – review & editing:** Zhiwei Zhou, Ping Xu.

## References

[1] Silvis SM de Sousa DA Ferro JM Coutinho JM. Cerebral venous thrombosis. Nat Rev Neurol. 2017;13:555–65. 28820187 10.1038/nrneurol.2017.104

[2] Qiu Z Sang H Dai Q Xu G. Endovascular treatments for cerebral venous sinus thrombosis. J Thromb Thrombolysis. 2015;40:353–62. 25771984 10.1007/s11239-015-1205-7

[3] Piazza G. Cerebral venous thrombosis. Circulation. 2012;125:1704–9. 22474313 10.1161/CIRCULATIONAHA.111.067835

[4] Ferro JM Aguiar de Sousa D. Cerebral venous thrombosis: an update. Curr Neurol Neurosci Rep. 2019;19:74. 31440838 10.1007/s11910-019-0988-x

[5] Gangat N Guglielmelli P Betti S. Cerebral venous thrombosis and myeloproliferative neoplasms: a three-center study of 74 consecutive cases. Am J Hematol. 2021;96:1580–6. 34453762 10.1002/ajh.26336 PMC9293093

[6] Thiele J Kvasnicka HM Orazi A. The international consensus classification of myeloid neoplasms and acute leukemias: myeloproliferative neoplasms. Am J Hematol. 2023;98:166–79. 36200127 10.1002/ajh.26751

[7] Wen H Jin D Chen Y Cui B Xiao T. Cerebellar venous thrombosis mimicking a cerebellar tumor due to polycythemia vera: a case report. BMC Neurol. 2021;21:225. 34134639 10.1186/s12883-021-02261-1 PMC8207742

[8] Ragurajaprakash K Senthilkumar R Sathish Prabu SS Madeswaran K Kiruthika P. Post-traumatic cerebral venous sinus thrombosis – institutional study and literature review. Interdiscip Neurosurg. 2022;27:101398.

[9] Harris L Townsend D Ingleton R. Venous sinus thrombosis in traumatic brain injury: a major trauma centre experience. Acta Neurochir (Wien). 2021;163:2615–22. 34218332 10.1007/s00701-021-04916-x

[10] Bhatoe HS. Dural venous sinus thrombosis after head injury. Neurol India. 2015;63:832–3. 26588613 10.4103/0028-3886.170063

[11] Covut F Kewan T Perez O Flores M Haddad A Daw H. Apixaban and rivaroxaban in patients with cerebral venous thrombosis. Thromb Res. 2019;173:77–8. 30481600 10.1016/j.thromres.2018.11.018

[12] Grangeon L Gilard V Ozkul-Wermester O. Management and outcome of cerebral venous thrombosis after head trauma: a case series. Rev Neurol (Paris). 2017;173:411–7. 28495232 10.1016/j.neurol.2017.03.025

[13] McLornan DP Hargreaves R Hernandez-Boluda JC Harrison CN. How I manage myeloproliferative neoplasm-unclassifiable: practical approaches for 2022 and beyond. Br J Haematol. 2022;197:407–16. 35191542 10.1111/bjh.18087

